# Non-Motor Symptoms: The Hidden Face of Parkinson’s Disease

**DOI:** 10.3390/cells15010042

**Published:** 2025-12-25

**Authors:** Carlo Cattaneo, Andrea Fabbrini, Daniele Belvisi, Flavia Aiello, Francesco Marchet, Giovanni Fabbrini

**Affiliations:** 1 Medical Department, Zambon SpA, 20091 Bresso, Italy; 2 Neurology Department, San Giovanni Hospital, 00153 Rome, Italy; fabbriniandrea89@gmail.com; 3 Department Human Neurosciences, Sapienza University of Rome, 00185 Rome, Italy; daniele.belvisi@uniroma1.it (D.B.); flavia.aiello@uniroma1.it (F.A.); giovanni.fabbrini@uniroma1.it (G.F.); 4 Neurology Department, IRCSS Neuromed, 86077 Pozzilli, Italy; francesco.marchet@uniroma1.it

**Keywords:** Parkinson’s disease, non-motor symptoms, quality of life, management

## Abstract

Non-motor symptoms (NMSs) of Parkinson’s disease (PD) were recognized by James Parkinson himself about 200 years ago and are now considered to be an integral part of PD, significantly contributing to the deterioration of patients’ quality of life. Although awareness of NMSs is growing, and several scientific societies now have dedicated non-motor study groups, the NMS burden is still the hidden face of PD and in most cases, clinicians’ views are focused on the motor symptoms alone. The literature was reviewed using several databases and scientific journals; this review provides a comprehensive description of the most common NMSs in PD, their clinical phenotype, social impact, diagnosis, and therapeutic management. Early recognition of these features may lead to more prompt and effective treatment and may help to better understand patients’ needs.

## 1. Introduction

Parkinson’s disease (PD) is a degenerative disorder of the central nervous system (CNS), typically characterized by a triad of motor symptoms, namely hypokinesia/bradykinesia, rigidity, and tremor, caused by a striatal deficiency of the neurotransmitter dopamine [1]. As such, current treatment of PD is based on the replacement of dopamine. While this can offer good control of motor symptoms, it does not halt the progression of neurodegeneration and consequent disability [1]. Globally, PD affects around six million people, and this number continues to increase due to numerous factors, including an ageing population, environmental factors, and a longer disease duration [2]. PD also presents a significant economic burden; in 2017, the estimated total medical cost attributable to this pathology was just over USD 25 billion in the United States alone [3]. As well as the cardinal motor symptoms of PD, there are also so-called non-motor symptoms (NMSs), including a wide variety of manifestations such as autonomic dysfunctions, hyposmia, sleep problems, neuropsychiatric symptoms, and cognitive impairment [4]. Patients often indicate that their NMSs are more difficult to manage than their motor problems [5]; the UK National Institute for Health and Care Excellence (NICE) recognizes NMSs and their management, as an important area of unmet need in PD [6]. However, a 2019 cross-sectional study found that up to 72% of people with PD do not report NMS to their healthcare professional [7]. The most commonly reported barriers to seeking help were acceptance of the symptoms, a lack of awareness that the symptom was associated with PD, and embarrassment to discuss some symptoms such as sexual problems or incontinence [7]. Clinicians often fail to identify NMSs during consultations, due to lack of time, a focus on motor aspects of PD, and the perception that no effective treatments are available [7]. The aim of this publication is to inform clinicians and other healthcare professionals about the key aspects of NMSs in PD, their pathophysiology, burden, economic costs, and treatment, and the importance of their diagnosis. As this is a narrative review, a systematic literature review was not conducted. A narrative literature search was performed using the PubMed, Cochrane, and Google Scholar databases to identify all relevant publications focusing on the epidemiology, clinical features and treatments of NMS. The study period was from 1990 to 2025. The following MeSH (medical subject headings) keywords were used: “Parkinson’s disease”, “treatments”, “neurotransmitters”, “genetic”, “non-motor symptoms”, “epidemiology “, “pathophysiology”, “clinical trials”. The terms were identified by reviewing titles and summaries. After exclusion of abstracts, duplicates, non-English articles, and those not addressing non-motor symptoms of Parkinson’s disease, we have included the publications that in our opinion are more relevant for this review, giving priority, whenever possible, to the recent literature. No new clinical or pre-clinical studies were performed by the authors, nor were patients recruited. No patient-specific efficacy or safety data were reported. Therefore, institutional review board/ethics approval was not required.

### 1.1. Epidemiology

NMSs are highly prevalent in PD patients: data of >2500 PD patients using the validated Non-Motor Symptoms Questionnaire indicated that almost all patients (>98%) reported up to eight different symptoms (Table 1) [8]; these results were confirmed in another group of 402 patients using the Movement Disorder Society NMS scale (Table 1) [9].

NMSs can also be classified according to their pathophysiology or drug therapy complications (Table 2) [9].

NMS heterogeneity may be related to the inclusion of patients of different age groups, duration of disease, and Hoehn and Yahr (HY) stage. Crosiers et al. revealed that the average number of NMSs was 6.5 in patients with disease duration <5 years and 10 in those with >15 years [10]. Hallucinations, urological, cardiovascular, and gastrointestinal symptoms are more frequent in older patients as well as in patients with HY stages 3-5, while younger patients or patients with HY stage ≤2 more frequently reported sleep problems, pain, anxiety, and depression [10]. Also, sex-related differences in the frequency and severity of NMSs have been reported by several studies [11]. Anxiety, sadness, depression, fatigue, dysphagia, constipation, and pain are more common in women, while men suffer more from sialorrhea, urinary dysfunction, hypotension, cognitive impairment, sleep behavior disorders, and daytime sleepiness [11]. Regarding impulse control disorders, men show compulsive sexual behavior while women are more likely to develop pathological shopping and “binge eating” [11]. Patients with PD also experience “non-motor fluctuations” (NMFs) which often develop in tandem with motor fluctuations, particularly during the “wearing off” periods, and female sex is a major risk factor for the development of NMFs [12]. Psychiatric symptoms, and in particular anxiety and depression, fluctuate more frequently and severely than other NMSs [13]. There are few studies investigating possible variations in NMSs among different ethnic groups, and their results are very preliminary; however, the current evidence suggests that NMS prevalence is high across all ethnic groups [14].

### 1.2. Genetics

An evolving area of research in PD relates to different genetic forms of PD and how they relate to non-motor signs. Although only a minority (5–10%) of PD cases are due to well-defined genetic causes, important clues about their pathology can be garnered from monogenic model diseases [15]. Genetic architecture contributes meaningfully to the expression of non-motor phenotypes in Parkinson’s disease, shaping both their timing and their domain specificity across olfactory, autonomic, sleep, neuropsychiatric, and cognitive circuits [16,17]. Building on this background, current evidence shows that monogenic and high-risk variants such as SNCA, LRRK2, PRKN, PINK1, DJ-1 and GBA shape non-motor trajectories in partly gene-specific ways. SNCA mutations often present earlier, progress more rapidly and carry prominent neuropsychiatric and autonomic features; LRRK2 exhibits heterogeneity with comparatively lower frequencies of severe cognitive decline in several series [18]. Recessive PRKN frequently shows a milder cognitive profile despite early motor onset; and heterozygous GBA variants, considered the most prevalent genetic risk factor for typical-phenotype PD, are associated with earlier onset and higher probabilities of cognitive impairment, visual hallucinations, and other neuropsychiatric features [19,20]. Grouping genes by predominant neuropathology helps interpret these correlations. Variants that drive Lewy-type α-synucleinopathy (classically SNCA and many GBA carriers) display wider cortical and limbic Lewy body involvement and more frequent cognitive and psychiatric manifestations. In this framework, GBA-associated PD is distinctive. Carriers display higher frequencies of both motor and non-motor complications, with earlier onset and a propensity toward cognitive decline and psychosis that tracks, at least in part, with variant deleteriousness [21]. By contrast, forms with less consistent Lewy pathology, such as LRRK2 and PRKN mutations, often show lower rates of dementia and hallucinations in aggregated reports, though clinical pathological confirmation remains limited [15]. These emerging data on the non-motor markers of genetic subtypes of PD may help refine anticipatory treatment strategies in the future. For example, this may include closer longitudinal surveillance of cognition, neuropsychiatric status and autonomic function in GBA and SNCA carriers. Genetics does not currently dictate a distinct pharmacological algorithm, but it can refine risk–benefit calculations and monitoring priorities across three axes: anticipating domain-specific non-motor trajectories, forecasting susceptibility to treatment-emergent complications, and informing thresholds for introducing adjunctive or alternative therapies [22]. For patients with variants that confer higher cognitive and psychiatric risk (most prominently, GBA) clinicians may favor slower titration of dopaminergic agents, vigilant screening for hallucinations and impulse control disorders, early consideration of non-dopaminergic symptomatic treatments for sleep, mood, and autonomic dysfunction, and proactive counselling of patients and caregivers on prognosis and safety. For those with profiles associated with lower rates of widespread Lewy pathology, such as many PRKN cases, standard dopaminergic strategies remain appropriate [23].

## 2. Timing of Symptom Presentation

NMSs often pre-date motor symptoms by 10–15 years, most notably depression or apathy, constipation, hyposmia, rapid eye movement (REM), and sleep behavior disorder (RBD) [24]. Other NMSs complicate the entire disease course (pain, fatigue) and especially the advanced stages (dementia, dysautonomia, psychiatric symptoms), as shown in the 20-year Sydney multicenter study [25]. There is growing evidence that the degeneration of non-dopaminergic neurons occurs before the dopaminergic neuron loss and the onset of motor symptoms [26]. By the time a patient shows the first clinically evident motor symptoms of PD, approximately 50% of the terminals and 30% of the nigral cells are lost, as estimated by postmortem data and using positron emission tomography (PET) imaging in a nonhuman primate model of PD [1]. This indicates that the pathological process begins at the nerve terminals and moves retrogradely, and that there is a substantial number of dopaminergic neurons that are dysfunctional but not lost [26]. This unique timing of symptom presentation in PD has prompted a redefinition of the disease led by the Movement Disorder Society (MDS) Task Force on the Definition of PD, giving rise to three distinct clinical stages [27], as follows:

- a preclinical phase, supported by molecular or imaging markers, but without clinical signs or symptoms of PD;
- a premotor phase (or prodromal phase), characterized by NMSs such as hyposmia and sleep behavior disorder;
- and the motor phase, often including NMSs such as pain, fatigue, and dementia.

## 3. What Causes NMSs in PD?

It is now recognized that multiple neurotransmitter pathways are impacted in PD, affecting areas that are not directly involved in motor control, as illustrated in Table 3 [28].

### 3.1. Dopamine Pathways Affected in PD

Dopamine is essential for the control of movement and also plays a key role in the control of systems involved in regulating pleasure, reward, sleep, and cognition. The nigrostriatal, mesocortical, and mesolimbic dopaminergic pathways are all affected in PD, contributing to depression, anxiety, pain, sleep disorders, cognitive impairments, and bladder dysfunction [28]. Dopaminergic therapies can also contribute to the development of some NMSs, such as hallucinations and daytime sleepiness [29].

### 3.2. Serotonergic Pathways Affected in PD

Serotonin neurons in the dorsal raphe nuclei project to the basal ganglia, the frontal cortex, and the limbic system [30]. The serotonergic system is thought to be involved in the control of various processes, including mood, emotion, and sleep; therefore, serotonergic dysfunction in PD has been directly linked to depression, anxiety, sleep problems, and visual hallucinations [30].

### 3.3. Noradrenergic Pathways Affected in PD

The locus coeruleus (LC) is the primary site of noradrenergic production in the brain, with neurons projecting out to numerous brain regions including the thalamus, the limbic system, forebrain, and the cortex [31]. Surprisingly, neuronal loss in the LC is greater (83%) than in the substantia nigra (78%) in PD, and the subsequent loss of noradrenergic innervation of the limbic system is associated with cognitive and neurobehavioral problems, including early cognitive dysfunction, dementia, depression, and anxiety [32].

### 3.4. Glutamatergic Pathways Affected in PD

Loss of nigral dopaminergic neurons and the subsequent striatal depletion of dopamine leads to glutamate hyperactivity in the basal ganglia [33]. Considering its widespread role in important central and peripheral processes, glutamate dysregulation (mainly hyperactivity) is implicated in cognition, depression, anxiety, sleep disorders and pain [33].

### 3.5. Cholinergic Pathways Affected in PD

Acetylcholine has several distinct functions in the brain, including arousal, attention, memory, and motivation. Cholinergic disruption in PD is known to contribute to cognitive deterioration, psychosis, sleep abnormalities, and altered olfactory function [34]. These symptoms have been specifically attributed to alterations in the cholinergic signaling in the striatum, and to degeneration of cholinergic nuclei, including the nucleus basalis and the pedunculopontine nucleus [35]. Cholinergic neurons also provide parasympathetic autonomic innervation to peripheral organs, and a reduced cholinergic tone contributes to urinary problems [35].

### 3.6. GABAergic Dysfunction Affected in PD

The γ-amino butyric acid (GABA) system is the main inhibitory neurotransmitter in the central, peripheral, and enteric nervous systems. A deficit of GABA may result in chronic dysfunctions such as sleep disturbances, hyposmia, hallucinations, and anxiety [36]. GABAergic dysregulation has been observed in PD patients in the basal ganglia post-mortem and in vivo with magnetic resonance spectroscopy [36].

Although multiple neurotransmitter systems are involved in Parkinson’s disease, they do not act in isolation. In particular, the dopaminergic and cholinergic systems interact at several levels, including the substantia nigra pars compacta, the striatum, and the ventral tegmental area [37]. This reciprocal interaction is supported by the co-expression of D1 and D2 receptors on cholinergic interneurons and by the presence of nicotinic receptors on dopaminergic terminals [38].

PET studies have shown an upregulation of the cholinergic system during the pre-motor phase of PD [39], and this appears to be inversely correlated with striatal dopaminergic binding [40].

Alterations have also been documented in the noradrenergic system, and these may begin even before dopaminergic degeneration. This is consistent with the known anti-inflammatory and neuroprotective effects of noradrenaline on dopamine neurons [41]. Similarly, early alterations in the GABAergic system have been reported [42]. However, unlike the noradrenergic system, GABAergic dysfunction usually progresses in parallel with dopaminergic degeneration, given the co-release of GABA by dopaminergic neurons [43]. A few studies have hypothesized, on the basis of studies in murine/rat models, that the glutamatergic alteration is secondary to the dopaminergic alteration, with a dynamic change in distinct glutamate receptor expression. Specifically, mGluR1 expression parallels the progression of PD, instead of mGluR5 [44]. A meta-analysis of PET studies underscored how the serotoninergic degeneration parallels PD progression, apparently in an independent manner from dopaminergic system, although the different ratio in speed degeneration between the serotoninergic and dopaminergic terminals is responsible for the levodopa-induced dyskinesia (LIDs) [45,46].

Different preclinical studies have documented the potential role of distinct categories of drugs in the treatment of PD, although most trials are nowadays focused on pathways of neuroinflammation and oxidative stress [47]. Pre-clinical studies are involved in the investigation of new drugs targeting specific mGluR with the aim of reduce LIDs and reduce dopaminergic degeneration [48], as well as for anti-noradrenergic drugs [41].

New pharmacological treatments selective for distinct muscarinic and nicotinic receptors and for serotoninergic receptors are being studied to treat dopamine-resistant symptoms and cognition [49,50].

Understanding what causes neuronal degeneration in the different neurotransmitter pathways remains the holy grail of PD research. One promising area of interest focuses on protein misfolding, a common feature of neurodegenerative disease [51]. In PD, misfolded α-synuclein forms insoluble aggregates, and Lewy bodies disrupt the functioning of many organelles, including mitochondria, endoplasmic reticulum (ER), and the Golgi apparatus (GA) [51]. Originally discovered in the substantia nigra, it is now known that Lewy bodies occur across different brain regions; this accumulation of α-synuclein outside of the nigrostriatal pathway is thought to contribute to the early onset of certain NMSs [51]. In the premotor phase of PD, misfolded α-synuclein has been noted in the enteric nervous system and is associated with the early symptoms of constipation and abdominal pain [52]. Misfolded and aggregated α-synuclein has also been observed in the olfactory bulb in early PD and linked to the premotor symptom of anosmia [52]. These observations led to a theory, spearheaded by Braak and colleagues, that a “bottom-up” pathological spread of misfolded α-synuclein underlies PD [53]. It has been postulated that α-synuclein-mediated neurodegeneration spreads in a caudo-rostral direction from the olfactory bulb, the enteric nervous system, the spinal cord, and the dorsal motor nucleus of the vagus nerve into the brain, affecting the different neurotransmitter populations before reaching the substantia nigra [53]. However, this theory may not apply to all patients. In fact, 7–8% of patients have been reported to have no α synuclein pathology in the dorsal motor nucleus [52]. Halliday and colleagues have shown that early-onset PD neurodegeneration and Lewy body deposition (predominantly limbic involvement) is different from that seen in later-onset disease (predominantly cortical and superior brainstem involvement) [54]. This leads to segregation into three distinct phenotypes: brainstem dominant, limbic dominant, and cortical dominant, each associated with different NMS [55]. Limbic dominant is mostly associated with symptoms of fatigue and pain, cortical dominant with cognitive symptoms and apathy, and brainstem dominant with sleep symptoms and dysautonomia [55].

## 4. The Burden of NMSs

NMSs seem to have a greater impact on patients’ quality of life (QoL) than their motor symptoms; in a large international cross-sectional study of 951 patients with PD, the highest burdens were noted in the pain, sleep, fatigue, mood, urinary, and gastrointestinal domains [56]. This effect was a direct consequence of symptoms and also indirectly due to consequent disability [57]. NMSs cause a significative burden not only for patients, but also for caregivers. The majority of care for patients with PD is provided by informal caregivers, not only offering physical and emotional support for patients but also playing a large economic supporting role and preventing early nursing home placement [58]. An online survey of people with PD and their caregivers found that care partners reported that NMSs were more challenging than motor symptoms (58% vs. 32%) [59]. NMSs commonly associated with caregiver burden are depression, anxiety, cognitive impairment, sleep disturbances, hallucinations, and psychosis [59]. Psychosis, in particular, causes great distress. A systematic review of UK studies found that caregivers had a comorbidity rate nearly five times greater than an age-matched population when the patients had psychiatric symptoms, with a comparable reduction in QoL over time affecting their social, psychological, and physical wellbeing [60]. The most frequent assessment scales used in these studies were the 39-item Parkinson’s Disease Questionnaire (PDQ-39) and the Non-Motor Symptoms Scale (NMSS). Finally, NMSs cause a large financial burden on patients, caregivers and healthcare system, and the economic costs are known to double with each Hoehn and Yahr stage [61]. Falls, dementia, and hallucinations are the major reasons for admission to hospital and residential care, while mood disorders and fatigue account for hidden costs such as sick leave, loss of productivity, and early retirement [62]. In the UK, the annual direct costs for patients living in full-time institutional care were estimated at about five times higher than in the home setting [63]. An Australian study found that the largest economic burden from the household perspective was the cost of informal care, which was estimated at AUD 12,548 per person over 12 months [63]. People with moderate-to-severe disease reported an average of 775 informal care hours annually [63]. The cost of formal care was 3.8 times higher for patients with 10 NMSs or more compared to those with 6–9 NMSs [64]. Moreover, women with PD receive less social and familiar care then men, and more frequently pay for a professional caregiver [11]. Clinically, the broad symptomatology and potential severity of NMSs requires multidisciplinary approaches, often with a dedicated team of around 15 different specialties [65]. Thus, through early identification of NMSs, physicians will be able to provide patients with better QoL, limiting the financial impact of PD.

### 4.1. A Focus on PD Nurses

With the convergence of numerous medical professionals for PD diagnosis and treatment, a specialist role was required to bridge the gap between this medical management and the unique personal needs of the patient [66].

In 1989 in the UK, the PD nurse specialist (PDNS) role was created to meet this need by providing information, education, and instruction, supporting the patient and caregiver in the promotion of self-management, supporting psychosocial care, specializing in diagnostic strategies and therapeutic nursing interventions, and promoting multidisciplinary collaboration.

Based on expert opinion from healthcare professionals, it is recommended that every person with PD could benefit from PDNS care, and the role is now common across Europe [66]. For example, in Sweden, the PDNS is the primary link to medical care for PD patients, evaluating changes in medical treatment and asking the patient for feedback on efficacy and side-effects [67]. Providing emotional support for both patients and caregivers is also an essential part of the work; as such, PDNS are often the first healthcare professionals to become aware about patients’ NMSs [68]. It is therefore important that the nurse is well-informed about the effects of these symptoms and any treatments that may be available [68]. PDNS care may improve patient wellbeing and quality of life and reduce anxiety and depression, saving money in the long term due to reductions in consultations with physicians and neurologists [68].

### 4.2. Tools for Assessing NMS

To effectively recognize and treat NMSs, clinically validated holistic tools have been developed and are now routinely used by physicians. The Non-Motor Symptoms Screening Questionnaire (NMSQuest) was developed by a multidisciplinary group of international experts in PD [69]. The questionnaire is a 30-item self-completed tool, with a yes/no response format, used to identify those symptoms requiring further evaluation during clinical consultation [69]. However, the questionnaire is only used for screening and does not grade symptom severity. Therefore, it cannot be used to assess treatment effects [69]. The Non-Motor Symptoms Scale (NMSS) is a 30-item rater-based scale that measures the severity and frequency of NMSs across nine dimensions in patients with PD at all stages of the condition, thus capturing the global burden for patients [70]. Importantly, the scale can be used in a clinical trial setting [70]. Other available tools include the battery of Scales for Outcomes in Parkinson’s disease (SCOPA) [71], investigating cognition, dysautonomia, sleep disorders and psychiatric symptoms, and the Movement Disorder Society Unified Parkinson’s Disease Rating Scale (MDS-UPDRS), which integrates motor and non-motor aspects of the disease [72]. There are other scales not specific for the assessment of NMS or grading only some symptoms, such as the Parkinson’s Disease Questionnaire–39 items (PDQ-39) [73], a self-administered quality of life scale including some questions related to cognition, mood, and pain, the King’s Parkinson’s disease Pain Scale (KPPS) [74] for PD chronic pain, the Parkinson’s Disease Sleep Scale (PDSS-2) and the Epworth Sleepiness Scale (ESS) for sleep disturbances [75,76], the Hamilton Depression rating scale (HAMD) and the Beck Inventory for depression [77,78], the Fatigue Severity Scale (FSS) and the 16-item Parkinson Fatigue Scale (PFS-16) for fatigue [79,80].

### 4.3. The Different NMS in PD

The symptoms can be broadly divided into five categories, as follows:

1. Pain and other sensory symptoms (olfactory dysfunction; changes in visual function);
2. Neuropsychiatric symptoms (depression; anxiety; apathy; cognitive impairment and dementia; psychotic symptoms, hallucinations and delusions; compulsive behaviors);
3. Sleep disorders (rapid eye movement sleep behavior disorder; insomnia; restless legs syndrome and periodic limb movements; excessive daytime sleepiness);
4. Autonomic symptoms (bladder dysfunction; gastrointestinal symptoms; neurogenic orthostatic hypotension; sexual dysfunction);
5. Fatigue.

### 4.4. Pain and Other Sensory Symptoms

#### 4.4.1. Pain

Pain affects between 40–85% of PD patients, and is considered one of the most bothersome NMSs, often more distressing than motor disability [81]. However, it is often underdiagnosed despite its substantial impact on patients’ quality of life; many patients also report poor management, and around 50% do not receive any analgesic prescription [81]. Pain is also one of the earliest symptoms of PD, sometimes present at the premotor stage [81]. There are various methods of classifying PD pain. The most widely accepted is the Ford classification, which distinguishes five different types of pain: musculoskeletal, dystonic, radicular/neuropathic, primary/central, and akathisia. These types also often overlap, further complicating diagnosis and treatment [82]. The Parkinson’s disease Pain Classification System (PD-PCS) is a new classification that has recently been published by a panel of specialists (including movement disorders experts, nurses, physiotherapists, and psychologists) and validated through an international, cross-sectional multicenter study [83]. The PD-PCS differentiates between pain related or unrelated to PD, and distinguishes three different types of pain: nociceptive, neuropathic, and nociplastic. The PD-PCS also provides a score that covers pain intensity, effect, and frequency [83]. The neurobiology of pain in PD is complex and appears to involve dopaminergic, serotonergic, noradrenergic, glutamatergic, and GABAergic neurotransmission [84]. In addition to motor functions, the basal ganglia are involved in the processing of nociceptive inputs; thus, nigrostriatal damage may at least partly account for the sensation of pain experienced in PD [84]. Furthermore, the serotonergic raphe nuclei and the noradrenergic locus coeruleus have a role in ‘gain setting’ for pain control and are impacted over the course of PD [84]. Changes in dopaminergic function in PD may also modulate sensory perception; for instance, dystonic and musculoskeletal pain occur more frequently in patients during ‘off’ periods, and pain thresholds are raised by dopaminergic medication [85]. Finally, peripheral sensory information and pain signals are transmitted to the spinal cord via primary afferent neurons, the majority of which are glutamatergic; upon noxious stimulation, glutamate is released from central terminals in the spinal cord [86]. Glutamate has also been implicated in the phenomena underlying chronic pain, including effects of allodynia and hyperalgesia [87]. Specific demographic and clinical factors have been associated with the variability of chronic pain in PD; age at onset, sex, ethnicity, and the presence of comorbid conditions can all shape the perception and experience of pain [86]. For example, females with PD may experience more severe or more frequent pain than males [88]. At the same time, people with younger-onset PD may report different pain patterns compared to those diagnosed later in life [89]. Genetic factors might contribute to these differences; for instance, the dopamine receptor D2 (DRD2) rs2283265 polymorphism has been associated with an increased risk of PD-related pain in females [90], while the CHRNA4 gene rs1044397 variant may influence PD onset age in females with chronic pain [91]. No drugs are specifically indicated for PD pain. Dopamine agonists can be useful for musculoskeletal pain, as can non-steroidal anti-inflammatory drugs and intra-articular corticosteroid injections, although in the MDS evidence-based medicine review these are considered “investigational” or “possibly useful” with “insufficient evidence” [84,92]. Deep brain stimulation (DBS), apomorphine, or adjustments of levodopa dosage are indicated for dystonic pain that occurs during wearing off, while botulinum toxin may be useful in localized dystonia, and amantadine may be useful for dystonic dyskinesia [84]. Antiepileptics (such as pregabalin and gabapentin), tricyclic antidepressants (TCAs) including amitriptyline, and serotonin and norepinephrine reuptake inhibitors (SNRIs) such as duloxetine are recommended for neuropathic pain [65]. The major limitation of the trials using these drugs is that pain was not the primary endpoint [84]. Glutamatergic neurotransmission is elevated during neuropathic pain; therefore, the use of glutamate modulators may help to reduce pain symptoms [93]. In a systematic review and meta-analysis, the greatest improvement of pain severity was seen with safinamide [94]. Safinamide reduced by 43.6% the total score of the King’s Parkinson’s disease Pain Scale, with significant improvements observed in in musculoskeletal, fluctuation-related, nocturnal, and radicular pain, and with a reduction of concomitant pain treatments by 26% [95]. Nevertheless, these results should be confirmed in double-blind controlled studies. In severe cases, the opioid drugs tramadol or tapentadol may be prescribed, while lidocaine or capsaicin patches or botulinum injections are used for peripheral neuropathies, although in this case, well-designed clinical trials are needed [96]. Data of 11.466 PD patients obtained from the French System of Health Insurance showed thatthe prevalence of analgesic drug prescriptions was higher in these patients compared to the general population (82% versus 77%) [97]. Also, chronic prescription (more than 90 days) was more prevalent in PD patients (33%) than in the general population (20%) [97]. Although these are the most frequently used drugs for pain in the disease, to our knowledge no controlled studies have evaluated their effect on PD pain. In addition to DBS, some non-pharmacological treatments may improve pain. High-frequency repetitive transcranial magnetic stimulation (rTMS) reduced musculoskeletal pain [98]. Acupuncture alleviates nociceptive pain, and physical activity, particularly Nordic walking, may be beneficial for back, hand, and leg pain, and yoga may be helpful for lower back pain [99]. These results should be considered carefully due to the lack of an active comparator.

#### 4.4.2. Olfactory Dysfunction

Hyposmia is clinically recognized as one of the earliest symptoms of PD and may precede the onset of motor symptoms by several years, although only half of patients with hyposmia or anosmia perceive their deficit, even when specifically asked [100]. A patient with hyposmia has a 10% increased risk of developing PD within 2 years of onset of olfactory dysfunction compared with asymptomatic relatives [100]. Thus, the symptom of hyposmia may represent an opportunity for early diagnosis and potential treatment before the onset of motor symptoms [100]. Hyposmia may arise from both orthonasal and retronasal olfactory dysfunction [101]. It is estimated that around 75% of people with PD have a poorer sense of smell compared with people of the same age; this includes both an increased olfactory threshold and a decreased ability to discriminate odors [101]. Bohnen et al. suggested that PD patients have selective hyposmia for some odors, rather than a general hyposmia [102]. They analyzed the smell scores of patients and healthy subjects; patients were unable to identify some odors compared to controls, and this failure correlates with reduced nigral dopamine activity [102]. As well as loss of smell, another recent unexpected finding is that patients with PD also have a unique ‘musky odor’ discernible by so-called ‘super smellers’ [103]. This unique scent results from several compounds, particularly hippuric acid, eicosane, and octadecanal being produced in higher than usual concentrations in the sebum of people with PD and it represents a potential biomarker for early diagnosis [103]. PD patients also display a reduced sniff airflow rate and volume, which influences their performance in olfactory function tests [102]. In addition to the olfactory deficits, olfactory hallucinations or phantosmia have been reported in PD patients. This phenomenon consisted of pleasant olfactory experiences such as fruits, fragrances or perfumed candles, and disappeared spontaneously in a temporal relationship with the development of motor symptoms [104]. The authors suggested that a local increase of dopamine activity may facilitate the occurrence of olfactory hallucinations, in the same way as dopaminergic agents induce visual hallucinations [104]. Another explanation may result from denervation-induced hyperexcitability of mitral cells in the olfactory bulb leading to spontaneous olfactory activity in the central olfactory system [104]. The reason why the sense of smell is impaired in PD is not completely understood; it has been suggested that it is related to early α-synuclein clumping in the olfactory bulb, visible before the loss of dopaminergic neurons in the substantia nigra [105]. According to the six-stage pathological process proposed by Braak and colleagues, Lewy bodies initially form in the olfactory bulb and anterior olfactory nucleus, producing olfactory dysfunction [53]. Lewy bodies, detected by α-synuclein immunoreactivity, were found in the olfactory bulb, the olfactory cortex and other brain regions related to olfaction (including the amygdala) in patients with PD [106]. Moreover, studies using magnetic resonance imaging revealed that, compared with healthy controls, individuals with PD showed an olfactory bulb that was flattened and shrunken [106]. In PD patients, the number of dopaminergic neurons in the olfactory bulb is twice that of healthy controls, probably due to a compensatory mechanism consequent to the nigrostrialtal dopamine deficit [106]. Hyposmia or anosmia have been reported in other disorders with abnormal synuclein, such as Lewy-body dementia and multiple system atrophy, suggesting a common pathogenic process [106]. Moreover, non-synucleinopathies such as vascular Parkinsonism, corticobasal degeneration, and progressive supranuclear palsy, tend to have intact olfactory function [107]. However, the presence of olfactory dysfunction in later stages of PD may also be linked to cholinergic denervation, cortical processing of olfactory inputs, the onset of cognitive deficits, and dementia [106]. Progressive hyposmia could therefore be used as a biomarker of cognitive decline in PD patients [105]. A 2010 study also found that individuals with PD have a loss of mitral cells and cells containing substance P in the olfactory bulb, and a reduction in the level of calcium-binding protein in this region [107]. While dopamine plays an important modulatory role throughout the olfactory system, dopaminergic pharmacotherapy has no discernible impact on smell loss [1]. Olfactory dysfunction is an important component of PD, but its pathological basis and relationship with the progression of the disease remains largely undefined.

#### 4.4.3. Changes in Visual Function

Parkinson’s disease is associated with several ocular disturbance, including blurred vision, diplopia, reduction of eye movements, impaired color discrimination, convergence insufficiency, and contrast deficits [108]. Up to 78% of patients are affected by such disturbances, and their incidence increases with disease progression [108]. Reduced blink rates can lead to dry eyes and hypomimia appearance; moreover, between 25% and 40% of patients experience visual hallucinations due to the presence of Lewy bodies in the occipital lobe and in retinal neurons, and to the loss of dopaminergic amacrine cells and of the regulatory role of dopamine receptors in the eye [108]. Treatment with dopamine agonists is associated with an increased risk of visual hallucinations, implying that dopaminergic signaling is involved in their generation, and visual hallucinations are predictive of cognitive decline in later stages of the disease [109]. Convergence insufficiency reduces binocular depth perception [108]. All these visual disturbances may negatively impact reading, driving, gait, balance (increasing fall risk), and daily activities, and they can significantly contribute to patients’ disability through their influence on cognitive and motor symptoms [108]. Reduced color discrimination is one of the visual hallmarks of PD, often emerging before motor symptoms, and may reflect neurodegenerative changes in the thalamus and posterior parietal lobe [110]. Patients show shortened chromatic fusion time, which indicates perceptual acuity for monochromatic contours, especially for light-blue and dark-green contours [110]. Innervation around the fovea (responsible for sharp central vision) is largely dopaminergic; postmortem studies of untreated PD patients showed decreased retinal dopamine concentrations compared with those treated with dopaminergic drugs [110]. The deposition of alpha-synuclein in amacrine and ganglion cells within the inner retina has been described [110]. Mutations in glucocerebrosidase are the most important risk factor for PD, and retinal thinning has been described in carriers of these mutations [110]. Many patients report blurred vision, which typically emerges at lower light levels during off periods [111]. Dopamine deficiency may reduce retina’s ability to differentiate spatially distinct stimuli, impairing contrast sensitivity, while during levodopa-induced dyskinesia, dopaminergic overstimulation may produce rapid fluctuations in contrast sensitivity, leading to blurred vision [112]. Artificial tears may alleviate dry eyes, botulin toxin injections can be used for blepharospasm, glasses or prisms for diplopia, whereas sustained release formulations of dopaminergic drugs may improve oculomotor symptoms during off periods, but should be monitored carefully due to the potential risk of hallucinations [113]. Anticholinergic drugs and amantadine should be avoided because may produce visual system side effects, such as mydriasis, photophobia, dry eyes, decreased accommodation, anterior angle closure, and blurred vision [113]. Care coordination among optometrists, ophthalmologists, occupational and physical therapists may be required [65].

### 4.5. Neuropsychiatric Symptoms

#### 4.5.1. Depression

Depression is a frequent neuropsychiatric symptom of PD, affecting up to 45% of patients with a substantial negative impact on their QoL, and can appear several years before the motor impairment [114]. Diagnosis can be difficult since some typical symptoms such as lack of expression, apathy, sleep disorders, loss of appetite, weight loss, lack of energy, and asthenia are also part of the clinical picture of PD [114]. It is a complex phenomenon that changes over the disease duration and may be a pathological consequence, a reaction to the associated disability, an independent separate comorbidity, or a combination of all three [114]. It has been hypothesized that depression in PD arises from degeneration of the mesocortical and mesolimbic dopaminergic neurons, disrupting serotonergic neurons in the midbrain and leading to dysfunction of the depression-related thalamic circuits [115]. Catecholamines may be also involved: depressed patients have a reduction in both dopaminergic and noradrenergic innervation in the locus coeruleus, thalamus, and in the limbic system [115]. The risk of suicidal behavior in PD patients is twice that in controls [116]. Selective serotonin reuptake inhibitors (SSRIs) such as fluoxetine, sertraline, citalopram, and paroxetine remain the first treatment of choice; a large clinical trial demonstrated the efficacy of the serotonin–norepinephrine reuptake inhibitor (SNRI) venlafaxine [117]. In the MDS evidence-based medicine review, venlafaxine was considered “efficacious” and “clinically useful,” whereas citalopram, sertraline, paroxetine, and fluoxetine were “possibly useful” [92]. Tricyclic antidepressant drugs affecting noradrenaline reuptake, such as amitriptyline and nortriptyline, may be effective in the treatment of PD-related depression. In the MDS evidence-based medicine review, they are classified “likely efficacious”; however, their use may be limited by side effects such as cognitive impairment and cardiac arrhythmia [92,117]. Pramipexole, a dopamine agonist, has been shown to improve depressive symptoms in PD patients and was considered “efficacious” and “clinically useful” in the MDS evidence-based medicine review [92,118]. Inhibition of monoamine oxidase enzyme increases the levels of biogenic amines (in particular dopamine) in the synaptic cleft and may enhance mood and motivation; nevertheless, rasagiline has shown no significant effects on depression in PD patients [119]. Growing evidence suggests that the glutamatergic system is directly involved in mood disorders in PD [120]. Glutamate levels are regulated by sodium-dependent glutamate transporter; mood stabilizing agents, like valproic acid and lamotrigine, modulate glutamate neurotransmission by blocking sodium channels [120]. Safinamide, a reversible MAO-B inhibitor and glutamate modulator, showed clinical benefits on mood fluctuations in patients with PD and improved emotional well-being, although further controlled studies are needed to confirm these results [121]. However, robust evidence for a good anti-depressant is lacking; the current evidence does not indicate one specific class of drugs, and the heterogeneity of contributory factors for PD depression should be considered by clinicians when prescribing a treatment [119]. The approach to the treatment of depression should be therefore multidimensional, comprising medications as appropriate, education about mood disturbances in PD, and emotional support [65]. Regarding non-pharmacological treatments, some beneficial effects on depression outcome measures have been reported with repetitive transcranial magnetic stimulation (rTMS), cognitive behavioral therapy (CBT) and deep brain stimulation (DBS) [122], though the MDS evidence-based medicine review highlights a need to better understand possible adverse events [92]. CBT in particular may be a first-line or add-on treatment for patients with mild depression and was classified “likely efficacious” in the MDS evidence-based medicine review [92]. In controlled, randomized clinical trials of CBT for PD depression, symptom severity improved by 56% compared with 8% for the control group [123]. Electroconvulsive therapy (ECT) may be considered for refractory depression, with caveats of side effects, limited trial evidence, and contraindication after DBS treatment [124].

Physical exercise, Tai Chi, yoga, acupuncture, and bright light therapy have shown beneficial effects with high variability, and the results should be confirmed by further studies. They are classified as “insufficient evidence” in the MDS evidence-based medicine review [92,122].

#### 4.5.2. Anxiety

Anxiety is very common in PD (prevalence 25–60%), but it does not fit the usual criteria for anxiety disorders, leading to a diagnosis of PD-specific anxiety whose manifestations are panic attacks, specific phobias (e.g., phobia of falling), social phobia, obsessive-compulsive disorder, or generalized anxiety disorder [125]. Anxiety is more frequent in females than in males and somatic symptoms include palpitations, shortness of breath, sweating, and dizziness [11]. Many symptoms can occur as a dopamine-deficit event related to ‘off’ periods and improve with appropriate dopaminergic therapy [125]. On the other hand, there are forms of anxiety, such as the fear of dying or going insane, that are independent of dopaminergic state and do not respond to dopaminergic drugs; these are more likely a reactive symptom to the diagnosis and progression of PD [125]. Anxiety can impact on the severity of the physical symptoms of PD.For example, patients report that episodes of anxiety can worsen pre-existing tremor or dyskinesia [126]. Interestingly, epidemiologic observations have found that patients with PD are at greater risk of developing anxiety before their PD diagnosis compared with age-matched controls, suggesting that anxiety may be an early non-motor marker of the condition [127]. Degeneration of subcortical nuclei and ascending dopamine, norepinephrine, and serotonin pathways within the basal ganglia–frontal circuits may be responsible for anxiety [127]. Positron emission tomography (PET) analysis has shown that the severity of anxiety in patients with PD is also inversely correlated with dopamine and noradrenaline-transporter binding in the amygdala, locus coeruleus, and thalamus [128]. These results suggest that anxiety in PD might be associated with a specific loss of dopaminergic and noradrenergic innervation in the locus coeruleus and the limbic system [128]. Trials of medications for PD-related anxiety are lacking [129]. The first step to treat anxiety is to optimize dopaminergic therapy, followed by the use of SSRIs, SNRIs, and tricyclics, which are to be monitored carefully due to their side effects [129]. Benzodiazepines, buspirone and pregabalin are sometimes prescribed, but with limited efficacy [129]. Benzodiazepines should be carefully used due to potential cognitive, balance, and sedating effects [129]. CBT, acupuncture, and Qigong may reduce anxiety symptoms [122]. CBT has large effects, maintaining improvements in situational anxiety, social anxiety, and avoidance behavior up to six months and reducing caregiver burden [130]. The results for acupuncture and Qigong should be confirmed by controlled trials [122]. The benefit of physical exercise for the treatment of anxiety in PD is much less convincing than for depression [122]. More research is needed to tailor treatment to the individuals’ needs and combined interventions may provide synergistic effects.

#### 4.5.3. Apathy

Apathy is one of the more common and debilitating neuropsychiatric disturbances in PD, present in 17–70% of patients, and consists of a loss of motivation, emotional indifference, and reduced desire to engage in activities [131]. The high heterogeneity in estimations of prevalence arises from the difficulty in distinguish apathy from other symptoms of PD such as bradyphrenia, depression, anxiety, cognitive impairment, or fatigue [131]. To address this difficulty, a more operational definition of apathy has recently been adopted; a diagnosis of apathy should be made if a quantitative reduction of goal-directed activity either in behavioral, cognitive, emotional, or social dimensions is observed in comparison to the patient’s previous level of functioning in these areas [132]. Apathy contributes to reduction in quality of life and increases distress among caregivers and patients’ dependency on care; moreover, apathy increases the risk of developing dementia, differentiating patients with intact cognition from those with cognitive impairment [132]. Motivation depends on subcortical structures linking the prefrontal cortex with the limbic system; disruption of these circuits through mesolimbic dopaminergic denervation, depletion of cholinergic neurons, and global serotonergic dysfunction results in feelings of apathy [133]. Decreased activity levels in the supplementary motor area and lesions in the basal ganglia and thalamus have also been implicated as underlying mechanisms of apathy [133]. Apathy and impulse control disorders have been considered as two faces of the same coin, caused respectively by hypo- and hyper-dopaminergic behaviors [134]. Dopaminergic contribution to apathy is supported by its onset after bilateral DBS; however, dopaminergic replacement therapy generally has a partial or no effect on apathy in PD [135]. The dopamine agonist piribedil showed some preliminary positive results, while rotigotine was ineffective; therefore, the role of other neurotransmitter systems involved in the normal functioning of the basal ganglia should be considered [136]. Piribedil was considered “likely efficacious” and “possibly useful” in the MDS evidence-based medicine review, while rotigotine was classified “unlikely efficacious” [92]. Substantial evidence implicates glutamine-to-glutamate ratio in the nucleus accumbens for the prediction of specific components of motivated behavior [137]. Safinamide, a glutamate modulator, significantly reduced scores on the Apathy Evaluation Scale and the 14-item Apathy Scale in 46.6% of clinically apathetic patients, but this result should be confirmed in large clinical trials [138]. The cholinergic system may have also a role in apathy; a multicenter, placebo-controlled trial with the anticholinesterase agent rivastigmine, administered via a transdermal patch, reported some improvements in apathy [139]. The MDS evidence-based medicine considers rivastigmine “efficacious” and “possibly useful”, but attention is required in its prescription due to potential adverse events [92]. The use of antidepressants for apathy is controversial. SSRIs have been related to increased apathy in PD, while the norepinephrine–dopamine reuptake inhibitor bupropion improved motivation, although this result should be considered preliminary [136]. One study using 5-hydroxytryptophan observed positive effects on depression but not on apathy [137]. Physical exercise (aerobic, dance, Nordic walking) and mindfulness may be useful but their benefit should be confirmed by controlled trials; exercise improves apathy through changes in goal-directed behavior and engagement in social interaction, and it improves mindfulness by targeting the emotional and cognitive domains [140]. There are currently no guidelines for managing apathy in PD; hence, there is an urgent unmet need to adequately explore treatments to improve this NMS.

#### 4.5.4. Cognitive Impairment and Dementia

Analysis of NMS Questionnaire data revealed that 46% of patients with PD experience cognitive problems with preserved function, known as mild cognitive impairment (MCI), at time of diagnosis, and 70–80% of patients will progress to PD dementia (PDD) within 15–20 years [141]. PD patients have a fivefold increased risk of developing dementia compared with their equivalent healthy age group [141]. People with MCI may experience difficulties with memory, language, thinking, or judgement that are greater than the cognitive changes expected with normal aging, whereas PDD is characterized by impairments in attention, executive, memory (especially retrieval rather than recognition) and visuo-spatial functions [142]. In a meta-analysis of 32 cohort studies, nine risk factors were found for PD cognitive impairment, including hallucinations, orthostatic hypotension, cerebrovascular disease, obesity, cardiac disease, alcohol consumption, smoking, and diabetes mellitus [143]. Neuroimaging studies revealed the presence of cortical and subcortical Lewy body with consequent atrophy, white matter hyperintensities, and disrupted cortico-striatal connectivity [142]. Low baseline levels of cerebrospinal fluid biomarker amyloid beta 1-42 (CSF Aß1-42) and p-tau and decreased Aß1-42:tau ratios may be predictors of cognitive decline in PD [144]. This observation is consistent with the one seen in Alzheimer patients with the greatest cognitive decline [144]. Another promising biomarker in neurodegenerative diseases is the neurofilament light chain protein (cNfL), which provides a sensitive measurement of neuroaxonal damage [145]. Recent studies have shown that higher cNfL is related to more severe PD symptoms, as measured by clinical scales, and shorter survival [145]. All these biomarkers may provide clinically useful prognostic information, particularly if combined with other risk factors for cognitive impairment in PD [143]. Cognitive decline is associated with significant impairments in quality of life, loss of independence, caregiver burden, and increased healthcare costs [142]. Given the heterogeneous neuropathological and neuropsychological nature of cognitive deficits, it has been hypothesized that there are two independent, partially overlapping syndromes in PD: a “frontal-striatal” network dysfunction present at the early stage of the disease, which is dopamine-modulated, leading to deficits in executive functions, working memory, attention, planning, and response inhibition; and “posterior cortical” degeneration associated with Lewy body formation and cholinergic loss, leading to dementia, characterized by visuospatial and memory deficits [146]. As such, dopaminergic drugs have little effect and can worsen psychosis and hallucinations associated with this dementia [146]. Loss of noradrenergic input from the locus coeruleus to cortical regions has also been noted in more cases of people with PD-related dementia compared with those without [147]. A multidisciplinary approach integrating pharmacological, non-pharmacological and psychosocial strategies is critical [66]. Few studies have addressed the treatment of PD cognitive impairment. The first step should be to discontinue drugs that can aggravate cognitive deficits, such as tricyclic antidepressants and benzodiazepines [148]. Cholinesterase inhibitors such as rivastigmine and donepezil are generally prescribed in common clinical practice; however, this class of drugs is associated with a higher rate of discontinuation due to adverse events. Rivastigmine was considered “efficacious” and “clinically useful” in the MDS evidence-based medicine review, while donepezil was classified as “insufficient evidence” [92,129]. Memantine and amantadine, two glutamate NMDA receptor antagonists, slightly improve cognitive dysfunction but are classified as “insufficient evidence” in the MDS evidence-based medicine review [92,148]. Finally, although a small, open-label pilot study of the SNRI atomoxetine found an improvement in cognitive function measures, these results have not been confirmed in other trials [149]. Non-pharmacological strategies include transcranial direct-current stimulation, physical exercise (aerobic or dance), Tai Chi or Qigong, and cognitive training, which are all classified as “insufficient evidence” by the MDS evidence-based medicine [92,150].

#### 4.5.5. Psychotic Symptoms, Hallucinations and Delusions

Psychosis is one of the major reasons for admitting PD patients into nursing homes and long-term care facilities, and it has been associated with worsened quality of life, caregiver burden, and increased mortality [151]. Prevalence of psychosis varies widely, being rarely reported in early stage of PD and ranging from 20–70% in advanced stages [151]. Manifestation of psychosis ranges from minor signs (mild illusions, vivid dreams) to more severe symptoms (visual hallucinations, paranoid delusions, and delirium) [151]. Visual hallucinations have been observed in up to 40% of patients with advanced disease and range from passing shadows in the periphery to recognizable people or animals. These often precede or accompany cognitive decline; as they become more vivid, the patient may lose insight into what is happening and start acting upon the hallucinations [152]. Visual hallucinations often precede or accompany cognitive decline and should be considered as a warning sign for dementia development and have also been reported in other neurodegenerative (e.g., dementia with Lewy bodies) or ophthalmological (e.g., Charles Bonnet syndrome) disorders [152]. The frequency of visual hallucinations varies from numerous times a day to weekly. They are most likely in the evening or at night and of brief duration [152]. Occasionally, auditory hallucinations also appear, and these can be a sign of depression [152]. Auditory hallucinations have sometimes been described as providing a “soundtrack” to visual hallucinations, for example, hearing people in a vision conversing [152]. Other less common types of hallucinations are tactile, olfactory, or gustatory hallucinations [152]. Tactile hallucinations have been described as the feeling of being touched by a person or involving contact with a small animal [152]. Paranoid delusions are less common than psychosis (occurring in 5% to 10% of patients with PD) but can be extremely distressing for both the patient and relatives, with common recurring themes of spousal infidelity or abandonment, intent of harm by strangers, or even by caregivers [153]. As with the other neuropsychiatric symptoms, psychosis in PD remains poorly understood. Neuroimaging studies with magnetic resonance showed gray matter atrophy and white matter abnormalities in parietal, temporal, and occipital regions of the brain, suggesting altered executive and cognitive functions, such as selective attention, cognitive flexibility and control, and emotional processing [154]. The pathophysiological processes underlying PD psychosis can be subdivided into intrinsic (derived from neurotransmitter dysfunction) and extrinsic causes (a result of the use of pharmacological agents) [154]. Intrinsic psychosis is thought to be caused by alterations in dopamine, serotonin, and acetylcholine systems involving subcortical projections, as well as synaptic and neuronal changes in limbic and cortical structures [154]. Visual hallucinations have been linked to the presence of Lewy bodies in the occipital lobe and in retinal neurons, and to the loss of dopaminergic amacrine cells and the regulatory role of D1 and D2 dopamine receptors in the eye [154]. Although they are often an adverse event of PD medications, neuronal degeneration of the pedunculopontine nucleus, locus coeruleus and dopaminergic raphe nuclei may be the origin [154]. Sleep disturbances commonly precede psychosis in PD. Acetylcholine is an important neurotransmitter in the control of REM sleep and some researchers have suggested a link between hallucinations and REM sleep behavior disorder [155]. It has been postulated that hallucinations might represent a narcolepsy-like state with intrusions of REM sleep into wakefulness [155]. During sleep, vivid dreams, nightmares, and night terrors occur more frequently in those patients with psychosis, and these phenomena may have similar underlying mechanisms to daytime hallucinations [155]. Various risk factors have been associated with the development of PD psychosis, including the use of dopamine agonists and a polymorphism in the cholecystokinin gene [156].

Management of psychosis is based on reduction or discontinuation of dopaminergic medication, in particular dopamine agonists, and of drugs that may have negative effects such as anticholinergics, benzodiazepines, phenothiazines and uro-spasmolytics [157]. Antipsychotics with dopamine antagonism, such as haloperidol, risperidone, and olanzapine, should not be given because they can worsen motor symptoms, while atypical antipsychotics blocking serotonin may be useful, in particular, clozapine which is the most efficacious but requires blood monitoring for the risk of agranulocytosis [157]. Clozapine was considered “efficacious” and “clinically useful” in the MDS evidence-based medicine review [92]. A further option is to use cholinesterase inhibitors such as rivastigmine or donepezil, to be monitored frequently due to their adverse events [157]. Pimavanserin, a serotonin 2A receptor inverse agonist, has obtained regulatory approval in the US for PD psychosis and is considered “efficacious” and “clinically useful” in the MDS evidence-based medicine review [92,158]. Psychosocial support for patients and caregivers is essential, while visual aids and art therapy may also be helpful [159]. More research is needed in this area to determine what is the most effective treatment for managing this problem.

#### 4.5.6. Compulsive Behaviors

Compulsive behaviors or impulse control disorders (ICDs) include a variety of repetitive, often rewarding, actions, from pathological shopping, eating, gambling, to hypersexuality and medication abuse [160]. These behaviors are problematic not only for the patients but also for the caregiver and are often not declared because they may be socially unacceptable, embarrassing, and a source of financial distress; therefore, physicians must specifically ask for their presence during patients’ visits [160]. Patients with ICDs and related disorders typically continue their addiction despite negative consequences. Any attempt to discontinue the behavior frequently leads to dysphoria, anxiety, and depression, similar to withdrawal symptoms after drug abuse [161]. Similar to the general population, it is believed that the impulsive component together with the feeling of joy and gratification may be responsible for the initiation of the addiction, while a more habitual and compulsive component may be the culprit of persistence [161]. ICDs occur in 14% of PD patients, sometimes having more than one compulsive behavior, with pathological gambling ranging between 3% and 8%, hypersexuality occurring in 2-8% and compulsive medication use in 3-4%. Therefore, physicians must specifically ask for their presence during patients’ visits [161]. Dopamine agonist use is one of the risk factors, although this can also occur in untreated subjects, suggesting that other factors related to cognitive process, personality traits, and psychiatric problems may play a role [160]. Other risk factors include younger age at disease onset, longer disease duration, motor fluctuations, male gender (for hypersexuality and gambling) or female gender (for pathological shopping), apathy, depression, and cognitive impairment [160]. Levodopa is mainly implicated in dopamine dysregulation syndrome, associated with medication overuse and punding, which is the urge to perform senseless activities repeatedly [161]. The proposed pathophysiology involves alterations in dopaminergic functions within the nucleus accumbens and ventral striatum and altered connections between striatal and associative/limbic cortical regions, potentially enhanced during the “on” state, confirmed by functional magnetic resonance imaging studies [162]. These alterations pinpoint the dysfunction of reinforcement learning in ICD, which is biased toward the overvaluation of reward and underestimation of risk [162]. ICDs and punding arise from partially distinct pathogenic mechanisms. ICDs reflect reward-based dysregulation of the mesocorticolimbic dopaminergic system driven by dopaminergic overstimulation [162]. Furthermore, patients with ICDs had lower dopamine D3 receptors; this led to a supersensitivity of the remaining D3 receptors, making these patients more vulnerable to addictive behavior [162]. In contrast, punding is not reward-based but is instead characterized by repetitive, stereotyped, habit-like behaviors. Its pathophysiology is thought to depend predominantly on maladaptive plastic changes within the striatum, resulting in impaired inhibition of motor stereotypies, due to chronic intermittent stimulation of D1–D2 receptors [163,164]. A balance of dopamine agonists dosage or a gradual reduction is recommended rather than a sudden or rapid discontinuation that may cause a withdrawal syndrome with panic attacks, dysphoria, dysautonomia, sleep disturbances and, in rare cases, symptoms resembling neuroleptic malignant syndrome [161]. Subthalamic DBS may help manage ICDs, but worsening of symptoms after surgery has also been reported; thus, further studies are needed to confirm the efficacy [161]. Naltrexone and clonidine have “insufficient evidence” according to the MDS evidence-based medicine review [92,161], which is also the case for non-pharmacological CBT treatment [73].

### 4.6. Sleep Disorders

Nearly all PD patients have sleep disturbances that usually start early, with a prevalence of 60-98% [165]. The more common are rapid eye movement (REM) sleep behavior disorder (RBD), insomnia, restless legs syndrome (RLS), excessive daytime sleepiness (EDS), and sleep-disordered breathing [165]. The pathogenesis of sleep disorders is multifactorial, but degeneration of central sleep regulation centers in the brainstem and thalamocortical pathways, with involvement of dopamine, serotonin, noradrenaline, and hypocretin pathways being the most important [165]. Other factors that may contribute to sleep disruption include motor symptoms, nocturia, anxiety, depression, and dopaminergic treatments. Sleep disturbances greatly affect the quality of life of both patients and caregivers [166].

#### 4.6.1. Rapid Eye Movement Sleep Behavior Disorder (RBD)

RBD is a parasomnia characterized by vivid and frightening dreams associated with elaborate motor activity, as the patients “act out” their dreams, and loss of muscle atonia during REM sleep [167]. Bed partners report on vocalizations and abnormal and sometimes violent movements that may cause injuries such as lacerations, ecchymosis and dislocations [167]. Diagnosis of RBD should be made via single-night PSG testing because clinical criteria alone have been shown to be only 33% sensitive [168]. RBD is thought to precede the onset of motor symptoms in over 40% of PD patients and RBD is a risk factor for cognitive impairment and psychosis in PD [167]. The pathological basis of RBD is still unclear; a hypothesis is that RBD arises because of degeneration of lower brainstem nuclei, consistent with Braak stages 1 and 2 [169]. Degeneration of the sublaterodorsal nucleus with direct and indirect projections to the spinal interneurons has been implicated, as well as involvement of the laterodorsal tegmental nucleus, perilocus coerulus region, nucleus reticularis magnocellularis, pedunculopontine nucleus, and ventrolateral reticulospinal tracts [169]. Sleep fragmentation may result, partly due to nighttime motor fluctuations; this can be mitigated by continuous drug delivery or by controlled-release formulations of L-dopa or dopamine agonists [170]. Use of clonazepam, melatonin, rivastigmine, and sodium oxybate may reduce involuntary nocturnal movements during sleep [170]. Clonazepam should be monitored due to potential side effects like sedation, confusion, and falls. Melatonin, rivastigmine, and sodium oxybate require further studies because the results are preliminary [170]. In a recent study, safinamide had a beneficial effect on patients’ sleep, as assessed by both clinical scales and PSG recordings, while rasagiline did not improve PSG sleep parameters nor clinical questionnaires. This result should be confirmed in larger studies [171].

#### 4.6.2. Insomnia

Difficulty falling asleep and difficulties maintaining sleep are both common in PD and risk factors include advanced disease and female gender [11]. Sleep maintenance problems and consequent sleep fragmentation can be caused by many different problems, such as nighttime akinesia, dystonia, and non-motor problems (nocturia, mood disorders, obstructive sleep apnea), and may be associated to daytime sleepiness [172]. Management of insomnia thus depends on a precise analysis of the cause. If the reason is mainly due to nocturnal PD symptoms, additional nighttime dopaminergic medications doses, preferably long-acting formulations, may be helpful [172]. For example, transdermal delivery of rotigotine can be effective in improving nocturnal motor disability, as rotigotine does not share the high-affinity D3 receptor profile of other dopamine agonists [172]. Rotigotine was considered “likely efficacious” and “possibly useful” in the MDS evidence-based medicine review [92]. When there is a suspicion that insomnia could be a side effect of Parkinsonian drugs, such as selegeline or dopamine agonists, it might be advisable to reduce their dosages or to change the treatment [173]. Antidepressants, anxiolitics, and melatonin have weak evidence for sleep insomnia and are considered having “insufficient evidence” in the MDS evidence-based medicine review [92,173]. Regarding non-pharmacological treatments, basic practices of sleep hygiene are part of standard management [150]. CBT and bright light therapy may also improve sleep efficiency, but new controlled trials are needed to confirm their efficacy [150].

#### 4.6.3. Restless Legs Syndrome and Periodic Limb Movements

Restless legs syndrome (RLS) and periodic limb movements are closely linked and are sensitive to dopaminergic treatments; 61% of PD patients reported a correlation between RLS and nighttime “wearing off” [174]. RLS is defined as an unpleasant feeling in a limb that appears or worsen when the patients is sitting or lying, mainly in the evening or at night, and may cause uncontrolled and sometimes potentially dangerous movements [174]. The relationship between PD and RLS is not completely clear, and their symptomatology may overlap [174]. RLS and periodic limb movements are other frequent causes of sleep disruption. The prevalence of RLS is estimated at 20% in PD patients, with an onset around 4.5 years after PD onset [175]. The pathophysiology of RLS and periodic limb movements is thought to be related to changes in mesocortical dopamine [175]. Continuous infusion of apomorphine resulted in significant reduction of nocturnal discomfort and decreased leg movements [176]. Long-acting levodopa, dopamine agonists, gabapentin, pregabalin, and intravenous iron can be considered for monitoring potential adverse events, while medications that can exacerbate symptoms, such as antidepressants, anticholinergics, and antihistaminergics, should be avoided [176].

#### 4.6.4. Excessive Daytime Sleepiness (EDS)

Excessive daytime sleepiness (EDS), defined as a debilitating trend to drift off to sleep, or rapid-onset sleep without any prior drowsiness, affects up to 60% of PD patients and can profoundly influence quality of life [177]. EDS can occur early in disease development, often predating diagnosis, and may be linked to cognitive impairment in the late stage of the disease [177]. Daytime somnolence is associated with poor concentration and memory and may lead to driving and/or occupational accidents [177]. The causes are multifactorial and include the disease process itself, nighttime sleep disruption, depression, and drug therapy with agents including antihistamines, dopaminergic therapies, anxiolytics, and selective serotonin reuptake inhibitors [178]. Haq et al. suggested a secondary narcoleptic phenotype in PD connected to degeneration of hypocretin-containing neurons in the hypothalamus [179]. Saper et al. proposed that a “flip-flop” switch is responsible for the sleep–wake cycle in which wake- and sleep-promoting neurons inhibit each other, resulting in stable wakefulness and sleep; disruption of wake- or sleep-promoting pathways results in behavioral state instability [180]. Dopaminergic dysfunction and neural degeneration have been suggested to destabilize the switch and its regulators, promoting rapid transition to sleep; dopamine agonists and levodopa could be involved [180]. EDS in PD patients is also associated with reduced spontaneous neural activity in the left angular gyrus and with reduced functional connectivity between the left angular gyrus and cerebellum [181]. EDS and sudden onset of sleep can increase the risk of serious injury, particularly if the patients drive a vehicle or operate machinery [177]. To improve these symptoms, exclusion/substitution of the suspected drugs can be tried. Treating patients with stimulants such as modafinil or caffeine is only partially efficacious, while anti-H3-receptor drugs solriamfetol and sodium oxybate seem more promising, but their efficacy should be confirmed in larger trials. These are considered having “insufficient evidence” in the MDS evidence-based medicine review [92,178]. Sleep-hygiene education and exposure to bright light may be helpful [150].

### 4.7. Autonomic Symptoms

Autonomic dysfunction in PD is common and debilitating, encompassing bladder, gastrointestinal, cardiovascular, and sexual problems and may precede motor features [182].

#### 4.7.1. Bladder Dysfunction

Bladder dysfunction is a common complaint in PD and is associated with poorer QoL, alteration of daily routine, falls, and admission to care, with a greater effect on emotional and social wellbeing on men than in women [183]. Urinary symptoms that predominate are storage symptoms (urinary urgency, frequency, and nocturia), suggestive of detrusor hyperreflexia (hyperactive bladder due to detrusor overactivity), classified as “irritative”, and voiding symptoms (incomplete emptying, slow and/or interrupted stream, terminal dribble, hesitancy, and straining), classified as “obstructive” [183]. Over 80% of PD patients experience these problems during the course of their illness [183]. In the PRIAMO study, the urinary dysfunction was reported in 57.3% of patients ranging from 37.7% in early PD (Hoehn and Yahr stage 1) to 89.8% in late stage (Hoehn and Yahr stage 4-5). Nocturia was the most prevalent (up to 86% of patients) followed by urgency (33-71%) and frequency (16-68%) [184]. These symptoms may lead to urinary incontinence and cause sleep disturbances [183]. Dopaminergic mechanisms play an important role in normal micturition control; dopaminergic neurons have both inhibitory and stimulatory effects on micturition, acting via D1 and D2-receptors, respectively [185]. Such neurons are particular abundant in the substantia nigra pars compacta (SNC) and the ventral tegmental area (VTA) of the midbrain and dysfunction of these mechanisms may lead to detrusor overactivity (DO) [186]. The most widely accepted theory is that basal ganglia inhibit the micturition reflex in the normal situation via D1 receptors, and cell depletion in the SNC in idiopathic PD results in loss of D1 receptor-mediated inhibition and consequently, DO [186]. Severity of urinary dysfunction may also correlate with the relative degeneration of the caudate nucleus, which receives dopamine-rich innervations from the SNC and VTA [186]. When treating urinary dysfunction, it is important to establish whether the symptoms are related to fluctuation; if so, adjustment of dopaminergic medication should be considered [186]. Levodopa showed both aggravation and alleviation of bladder symptoms, worsening detrusor overactivity during bladder filling, but facilitating voiding through an effect on detrusor contractility; apomorphine improved voiding efficiency by increasing urine flow and reducing post-void residual urine volume, although its effect on detrusor muscle may vary [186]. Antimuscarinics, in particular, trihexyphenidyl and oxybutynin, are the most routinely used class of drugs to treat overactive bladder symptoms in PD. However, these treatments should be used with caution in patients experiencing hallucinations and cognitive decline [186]. Moreover, blocking M2 and M3 receptors can lead to dry mouth and constipation. The beta-3 adrenergic agonist, mirabegron, and intravesical botulinum toxin injections can be also considered, having improved the overactive bladder symptoms with mild side effects [187]. In a retrospective study, safinamide improved urinary symptoms (measured through the SCOPA Autonomic Urinary scale), as well as urgency, incontinence, frequency, and nocturia. These results should be confirmed in controlled trials [188]. Non-pharmacological treatments include intermittent catheterization and pelvic floor therapy [189]. Finally, patients should be referred to a urologist for further investigation if persistent urinary symptoms are suspected to be related to bladder outlet obstruction.

#### 4.7.2. Gastrointestinal Symptoms

Gastrointestinal disorders are present in more than half of patients with PD, including salivary excess (sialorrhea), dysphagia, nausea/gastroparesis, and bowel dysfunction (constipation, defecatory dysfunction) and can cause serious complications, such as weight loss [190]. Gastric dysfunction may impair drug absorption and contribute to motor fluctuations; moreover, antiparkinson medications themselves exacerbate the symptoms [4]. Gastrointestinal function is impaired primarily due to disruption to both extrinsic and intrinsic innervation of the gut, partly caused by accumulation of Lewy bodies and alpha-synuclein deposits in the dorsal motor nucleus of the vagus nerve, sacral parasympathetic nuclei, sympathetic ganglia, and the enteric nervous system (ENS), with consequent microbiome changes and inflammation [191]. The ENS plays a key role in the generation and coordination of antral contractions and peristalsis and in the regulation of gastric emptying [191]. The effects of PD on skeletal muscle function in the oropharynx, anorectum, and pelvic floor also contribute to the problem [191]. Multiple neurotransmitters and neuromodulators (for example, acetylcholine, serotonine, dopamine, noradrenaline, vasoactive intestinal peptide and nitric oxide) control gastrointestinal function. Thus, it is unlikely that there is a single pharmacological basis responsible for the symptomatology observed in PD [191]. Sialorrhea (excessive build-up of saliva in the mouth) affects 70–80% of patients and is generally observed in the late stages of the disease and during ‘off’ states. Saliva accumulation is caused by poor bolus formation and reduced frequency and efficiency of swallowing [192]. Drooling (involuntary spillage of saliva from the mouth) can affect eating and speech and feeding and cause social embarrassment. Sialorrhea and drooling are mainly due to dysphagia causing less frequent and efficient swallowing and are often compounded by the tendency to keep the mouth open (facial hypomimia) [193]. Chewing gum or sucking on hard candy, which converts swallowing into a more conscious action, may provide temporary improvement and relieve drooling in some social situations [193]. Salivation is mediated primarily through parasympathetic (cholinergic) innervation of the salivary gland [193]. Locally administered anticholinergic drugs may be helpful, but their use should be limited to short periods because they can aggravate constipation or cause dry mouth, urinary retention and cognitive impairment [193]. Sublingual atropine drops, ipratropium spray, and botulinum toxin injections into salivary glands (except if there is esophageal dysmotility) may decrease saliva production without systemic adverse events. Only botulinum toxin was considered “efficacious” and “clinically useful” in the MDS evidence-based review [116,193]. Dysphagia affects from 30% to 80% of patients [190]. Although dysphagia worsens over time, the first signs can occur in the early stages, when the disease is often not yet detected. The risk of dysphagia in PD patients is three times that in control individuals [194]. Dysphagia for pills is an often-overlooked aspect of dysphagia in PD and may be evident in close to 30% of patients, particularly those with more advanced disease [193]. The oral, pharyngeal and esophageal stages of swallowing may be affected; stiffness and bradykinesia may slow oral transit time and residual food may become stuck inside the mouth [194]. Pharyngeal dysmotility can lead to increased risk of aspiration [194]. As a result, patients with this dysfunction are subject to malnutrition, dehydration, and increased mortality due to aspiration pneumonia [194]. There is no standard drug treatment for dysphagia; based on the literature, it is recommended to optimize the timing of administration of dopaminergic therapies to allow patients to consume meals in their best “on” state [195]. Among pharmacological options, botulinum toxin injections in the cricopharyngeal muscle may improve upper oesophageal sphincter relaxation during swallowing [195]. Non-pharmacological methods include chin-down swallowing, encouraging patients to eat slowly and use of thickened liquids [195]. Continuous monitoring of patients’ diet and nutritional care by clinical nutritionists reduces the risk of dehydration and malnutrition [195]. Nausea is often caused by dopaminergic medications but may develop without antiparkinsonian drugs due to impaired or delayed gastric emptying (gastroparesis), corresponding to decreased stomach motility, which can affect gut transit [190]. In addition to nausea, chronic problems with gastric emptying can cause a sensation of fullness, abdominal pain, and bloating [190]. Gastroparesis is characterized by impaired gastric emptying. It is present in nearly all PD patients and appears in the early stages of the disease [193]. Patients should be encouraged to eat small but frequent low-fat meals [4]. Gastroparesis may interfere with drug absorption and bioavailability [196]. Oral levodopa is absorbed in the proximal small intestine; slow gastric emptying delays levodopa delivery to the small intestine, increasing pre-systemic decarboxylation and resulting in reduced absorption and efficacy [196]. This may in turn be the cause of motor fluctuations; various formulations of levodopa and other anti-Parkinson drugs have been proposed to overcome this problem, such as controlled-release, subcutaneous, liquid, patch, and inhaled formulations [196]. On crossing the blood-brain barrier, domperidone, a dopamine-2-receptor antagonist, improves gastric emptying and drug absorption, but the dosage should not exceed 30 mg daily due to the risk of high dosages inducing prolongation of the QT interval and ventricular arrhythmia [197]. Domperidone was considered “likely efficacious” and “possibly useful” in the MDS evidence-based review [92]. Antiemetics with dopamine-blocking properties (e.g., metoclopramide, prochlorperazine) should be avoided [197]. Serotonin 5-HT4 receptor agonists stimulating acetylcholine release, such as cisapride and mosapride, have prokinetic properties, but their use is limited by their cardiotoxicity [197]. Mirtazapine and botulinum toxin injection may be useful in improving gastric emptying processes, but they have not been formally studied in PD [197]. Constipation is the most common bowel dysfunction, affecting around 50% of patients, and may precede the appearance of motor symptoms by several years [198]. The severity and rate of occurrence of constipation correlates with disease severity [198]. The dorsal motor nucleus of the vagus is important in autonomic control of the bowel, and pathological change in this area occurs early in the development of PD [198]. The pathophysiologic basis for reduced bowel movement frequency is decreased colonic smooth muscle and phasic rectal contractions, with consequent prolonged colon transit time; the average colon transit time in PD patients is more than twice that of healthy subjects [198]. Sigmoid volvulus is a potentially under-recognized serious complication of constipation in PD [198]. Management includes lifestyle and dietary modifications such as increasing physical activity and the consumption of fiber (e.g., psyllium) and fluids [199]. Probiotics have been suggested to have favorable effect in improving constipation based on the changes of gut microbiome [199]. Fiber and probiotics are considered “efficacious” and “clinically useful” in the MDS evidence-based review [92]. A stool softener such as docusate may also be helpful, as well as osmotic laxatives (for short-term use), prokinetic agents, and lubiprostone (a chloride channel activator), though long-term studies are needed [199]. Lubiprostone has shown the best efficacy in PD, with nausea as a potential side effect; it is “likely efficacious” and “possibly useful” in MDS evidence-based review [92]. Fecal microbiome transplantation for the treatment of constipation in PD is a source of interest and promise, but definitive studies are still lacking [199]. Defecatory dysfunction may be present in up to 70% of PD patients and may be evident in both early and advanced PD, resulting in pain, excessive straining and incomplete elimination [200]. Formal studies of potential treatment modalities have been rare [200]. There are no specific treatments; possible therapies include biofeedback training to relax muscles, abdominal massage, botulinum toxin injections into the puberorectalis muscle, and apomorphine injections, but these strategies should be confirmed by controlled trials [200].

#### 4.7.3. Neurogenic Orthostatic Hypotension

Blood pressure dysregulation is the most frequent cardiovascular symptom in PD, particularly neurogenic orthostatic hypotension (OH) and neurogenic hypertension in the supine position (SH) [201]. Management of symptomatic OH and SH can be challenging as treating one usually aggravates the other [201]. OH is the most common manifestation with a reported frequency of 30–58%; this prevalence increases with age and disease duration [201]. OH is highly disabling, increasing fall risk and decreasing independence [202]. Light headedness, blurry vision, dizziness, and feeling faint are easily recognized as caused by OH; other less specific symptoms include tiredness, cognitive impairment, dyspnea, neck and shoulder discomfort, or angina [202]. Symptoms are due to tissue hypoperfusion as a result of OH, defined as a sustained fall in blood pressure of ≥20 mmHg systolic or 10 mmHg diastolic when moving from supine to standing [202]. Normally, vasoconstriction maintains blood pressure in the standing position: standing up unloads the baroreceptors, which triggers norepinephrine release from sympathetic post-ganglionic nerves, causing vasoconstriction [203]. In patients with PD, this compensatory mechanism is absent or attenuated [203]. Plasma norepinephrine, a marker of sympathetic neuronal integrity, is lower in PD patients with OH; imaging and neuropathological data show that post-ganglionic peripheral sympathetic neurons innervating the myocardium and sympathetic fibers innervating blood vessels are functionally affected due to α-synuclein deposits and fiber loss [203]. This results in impaired norepinephrine release and defective vasoconstriction upon standing, causing the fall in blood pressure [203]. Consensus guidelines for the treatment of OH state that the goal is not to normalize standing blood pressure, but to reduce symptom burden, improve QoL, and reduce morbidity and mortality [204]. The first step to manage OH is to correct aggravating factors (eliminating unnecessary diuretics, vasodilators, and drugs that block norepinephrine release/activity at the neurovascular junction), investigate and treat anemia, and adjust the doses of levodopa and/or dopamine agonists [204]. Non-pharmacological strategies include reducing or stopping caffeine and alcohol intake, due to their diuretic and vasodilator effect, avoiding sugar beverages, increasing fluid intake, physical exercise, and salt intake (sodium-enriched diet), eating smaller, more frequent meals, and reducing carbohydrates in case of postprandial hypotension, elevating head position during sleep, and using support stockings or abdominal binders [204]. Drug therapies include fludrocortisone, or increasing peripheral vascular resistance with midodrine, droxidopa, or norepinephrine transporter inhibitors (atomoxetine) [204]. Of these, only droxidopa was considered “efficacious” and “possibly useful” in the MDS evidence-based review, while the other drugs should be monitored due to their side effects such as lower extremity edema, heart failure, electrolyte abnormalities, and supine hypertension [92].

#### 4.7.4. Sexual Dysfunction

Sexual dysfunction is frequently experienced by PD patients, with a prevalence from 36% to 80%, and is often poorly discussed or investigated in clinical evaluations, due to a lack of standardized assessment tools for sexual function [205]. Over two-thirds of male patients have erectile dysfunction (ED) while 70% of female patients have decreased libido [180]. Moreover, women with PD are more likely to have anxiety or inhibition during sex, vaginal tightness, and involuntary urination [205]. Other aspects of sexual function, such as orgasmic function, sexual desire, intercourse satisfaction, and overall satisfaction, are also impaired [205]. Sexual dysfunction occurs in young and elderly patients of both genders, at the beginning of the disease and throughout its course, and it can even be a premotor PD sign [205]. Impaired sexual function in PD patients may not arise solely from the neurodegenerative process affecting the autonomic nervous system [205]. Psychological issues (lowered self-esteem, depression, anxiety, apathy, relationship problems), fatigue, and pain may contribute substantially [205]. The use of SSRIs and tricyclics for comorbid depression, antihypertensive medications such as ß-blockers, or advanced disease stage may also contribute to the development of sexual dysfunction [205]. The exact pathophysiology of sexual dysfunction is not fully known; it has been hypothesized that disturbances of the mesocortical and mesolimbic dopaminergic pathways may be involved [206]. Libido and erection are thought to be regulated by the hypothalamus, particularly the medial preoptic area [206]. Dopamine may help to facilitate sexual behavior; in the paraventricular nucleus, dopamine activates oxytogeneric neurons that project to the hippocampus, medulla oblungata, and spinal cord, all of which play important roles in sexual motivation and reward [206]. Dopamine agonists such as apomorphine, ropinirole, and pergolide mesylate may be helpful in inducing erection; however, dopamine agonists may induce impulse control disorders, including hypersexuality [207]. The phosphodiesterase-5 inhibitor sildenafil is effective for treating erectile dysfunction but standing and lying blood pressure must be measured before prescribing this agent for potential orthostatic hypotension induction or deterioration [208]. Moreover, sildenafil cannot be given with nitrate therapy for cardiac disorders [208]. Sildenafil was considered “efficacious” and “clinically useful” in the MDS evidence-based medicine review [92]. Testosterone deficiency has been reported in men with PD, but the efficacy and long-term safety of testosterone replacement for the treatment of sexual dysfunction is unproven [208]. Deep brain stimulation (DBS) in the subthalamic nucleus may lead to improvement of sexual dissatisfaction, improving motor symptoms, however there are reports of the development of transient mania with hypersexuality following DBS [208]. Lubrication agents and systemic or local hormonal replacement therapy may help women with decreased libido; sex and behavioral therapy may also be helpful for addressing the psychosocial and relationship aspects of sexual dysfunction [207].

### 4.8. Fatigue

Fatigue in PD is a subjective sensation of profound tiredness, lack of energy, and exhaustion, contributing to restriction in daily activities and social activities and associated with nonrestorative rest [209]. Although fatigue often coexists with apathy, anxiety, depression, and sleep disorders, it is an independent symptom [209]. Up to 70% of the PD population experience fatigue, which may emerge at the early stages of the disease or in the premotor phase before motor symptoms and can be divided into mental or physical fatigue [210]. Physical fatigue is a lack of energy to perform physical tasks despite personal ability and motivation. Mental fatigue refers to the cognitive effects experienced during and after prolonged periods of activities requiring sustained concentration, such as driving in heavy traffic [210]. Despite its relevance, its pathophysiological mechanisms are still not fully understood. Several hypotheses suggest a multifactorial aetiology involving dysfunction in dopaminergic and non-dopaminergic pathways, neuroinflammation, genetic predispositions, and metabolic dysregulation [211]. Patients with fatigue have abnormalities in the prefrontal cortex, reduced perfusion in the frontal lobe and dysfunction in the connections between the hypothalamic–pituitary axis and the basal ganglia [211]. Dopamine is only partially involved in the pathogenesis of fatigue; other neurotransmitter systems should be considered, in particular, serotonin and glutamate [212]. A study published by Pavese et al. showed a significant reduction in serotonin reuptake transporter binding in the caudate nucleus, putamen, ventral striatum, thalamus, cingulate, and amygdala of PD patients with fatigue compared to those without [213]. Glutamatergic dysregulation is another key factor. Glutamate hyperactivity is implicated in excitotoxicity, oxidative stress, and neuronal death and contributes to the development of chronic inflammation, triggering the release of pro-inflammatory cytokines such as interleukin-6 and tumour necrosis factor alpha [214]. High levels of these cytokines have been observed in patients with severe fatigue; therefore, targeting glutamatergic pathways to mitigate neuroinflammation may represent a promising therapeutic approach for alleviating symptoms [214]. Recent studies showed a significant association between elevated levels of cerebrospinal fluid alpha-synuclein and fatigue in PD patients, probably due the activation of pro-inflammatory cytokines released in the microglial cells mediated by alpha-synuclein [215]. Versace et al. reported an increase in fatigue due to a reduction in GABAergic inhibition in the primary motor cortex [216]. Genetic factors also affect the fatigue mechanism, as several mutations have been linked with fatigue in patients with PD, such as glucocerebrosidase beta mutations [217] and the leucine-rich repeat kinase 2 (LRRK2) G2385R variant [218]. Management of fatigue requires a complex approach, including pharmacological, non-pharmacological, and lifestyle interventions [219]. Optimization of pharmacotherapy may alleviate fatigue. Levodopa and intrajejunal levodopa infusion may improve physical fatigue in PD patients; dopamine agonists, apomorphine, and selegiline have controversial effects in fatigue management, while rasagiline was found to decrease the fatigue scale score in PD patients [220]. Some studies support safinamide’s role in reducing PD-related fatigue, possibly due to its effect on glutamate regulation [221]. Modafinil and methylphenidate, two stimulant agents, may reduce physical fatigue, although the MDS evidence-based medicine review considered these results as “insufficient evidence” [92]. Doxepin (a tricyclic antidepressant) was associated with improvement in general fatigue (both physical and mental), while caffeine and memantine had no effect [220]. Bilateral subthalamic deep brain stimulation has shown potential benefits for some non-motor symptoms including fatigue [222]. Non-pharmacological strategies, such as structured exercise programs (in particular Nordic walking), CBT, dance therapy, and sleep optimization, have shown benefits in reducing fatigue severity [223]. Additionally, lifestyle modifications, including maintaining a balanced diet, managing stress, and ensuring proper hydration, can help improve energy levels [224].

## 5. Conclusions

In addition to motor symptoms, NMSs are now recognized as extremely frequent and important components of PD that can appear during all stages of the disorder, including the premotor stage. NMSs result from the impairment of multiple brain neurotransmitter systems and are a major determinant of health-related quality of life and progression of overall disability, emphasizing the need for early detection and awareness.

Despite their frequency and impact and the development of several validated scales assessing their severity, NMSs continue to be under-reported and inadequately treated. There is a need for more clinical trials considering NMSs as outcome measures. One of the major therapeutic challenges for PD is the development of effective symptomatic interventions for non-motor features. While promising, the data regarding the pharmacological and non-pharmacological therapies currently available for NMS remain heterogeneous and are often based on non-controlled or exploratory trials. Further well-designed, double-blind studies are needed to establish the role of these therapies across the entire non-motor spectrum. The growing interest in the identification of potential biomarkers, the effort to detect pre-motor phases, new disease-modifying therapeutic studies, and the use of digital tools and/or AI could provide a window of opportunity for the treatment of NMSs, their early diagnosis and the understanding of the role of dopaminergic and non-dopaminergic changes in their evolution.

With the necessary knowledge and expertise, clinicians should be able toaccurately detect NMSs in PD in a timely way and implement appropriate interventions to mitigate their impact.

## Figures and Tables

**Table 1:** Frequency of occurrence of the eight most common non-motor symptoms.

Non-Motor Symptoms	NMS-Q Mean (%)	MDS_NMS Mean (%)
Urinary problems	74%	68%
Pain	65%	65%
Fatigue	64%	65%
Sleep problems	62%	63%
Constipation	48%	48%
Cognitive impairment	46%	48%
Depression/apathy	43%	39%
Excessive drooling	35%	33%

**Table 2:** Clinical classifications of NMSs of PD.

Related to the disease process or pathophysiology	Dopaminergic, e.g., depression, anxiety, dystonic pain and sleep disorders
	Non-dopaminergic, (cholinergic, serotonergic, noradrenergic, glutamatergic, and mixed) e.g., hallucinations, urinary problems, cognitive impairment, neuropathic pain
Related to non-motor fluctuations	Present only during off periods, e.g., mood disorders, impaired concentration
	Present during on periods and worse in off e.g., pain, fatigue, constipation
Related to drug therapy for PD	e.g., hallucinations, impulse control disorders, excessive daytime sleepiness
Genetically determined	Dementia or MCI in patients with glucocerebrosidase mutation
	Depression and sleep disorders in patients with LRRK-2 mutation

NMSs: non-motor symptoms; PD: Parkinson’s disease; MCI: mild cognitive impairment; LRRK-2: leucine-rich repeat kinase 2.

**Table 3:** Brain regions and neurotransmitters implicated in NMSs.

NMS	Brain Region	Neurotransmitter					
		DA	SE	NA	GLU	ACE	GABA
Hyposmia	Olfactory bulb and amigdala					√	√
Hallucinations	Occipital cortex	√	√			√	√
Pain	Basal ganglia, locus coeruleus, raphe nucleus, amygdala, thalamus	√			√		
Anxiety	Limbic area including amygdala	√	√	√	√		√
Depression	Limbic and cortical areas	√	√	√	√		
Early cognitive dysfunction	Frontal cortex	√		√	√	√	
Dementia	Temporal, parietal and occipital lobes			√		√	
Sleep disturbances	Hypothalamus and reticular formation	√	√		√	√	√
Bladder hyperreflexia	Basal ganglia	√				√	

NMS: non motor symptom; DA: dopamine; SE: serotonine, NA: noradrenaline; GLU: glutamate; ACE: acetylcholine; GABA: γ-amino butyric acid.

## Data Availability

Data sharing is not applicable as no datasets were generated, used, or analyzed for this article.
